# RNASE2 Mediates Age-Associated B Cell Expansion Through Monocyte Derived IL-10 in Patients With Systemic Lupus Erythematosus

**DOI:** 10.3389/fimmu.2022.752189

**Published:** 2022-02-21

**Authors:** Yantong Zhu, Xiaojun Tang, Yang Xu, Si Wu, Weilin Liu, Linyu Geng, Xiaolei Ma, Betty P. Tsao, Xuebing Feng, Lingyun Sun

**Affiliations:** ^1^ Department of Rheumatology and Immunology, The Affiliated Drum Tower Hospital of Nanjing University Medical School, Nanjing, China; ^2^ Division of Rheumatology and Immunology, Department of Medicine, Medical University of South Carolina, Charleston, SC, United States

**Keywords:** systemic lupus erythematosus, age-associated B cells, ribonuclease A family member 2, interleukin 10, monocytes

## Abstract

Systemic lupus erythematosus (SLE) is characterized by the production of pathogenic autoantibodies. Ribonuclease A family member 2 (RNase2) is known to have antiviral activity and immunomodulatory function. Although RNASE2 level has been reported to be elevated in SLE patients based on mRNA microarray detection, its pathologic mechanism remains unclear. Here, we confirmed that RNASE2 was highly expressed in PBMCs from SLE patients and associated with the proportion of CD11c^+^T-bet^+^ B cells, a class of autoreactive B cells also known as age-associated B cells (ABCs). We showed that reduction of RNASE2 expression by small interfering RNA led to the decrease of ABCs *in vitro*, accompanied by total IgG and IL-10 reduction. In addition, we demonstrated that both RNASE2 and IL-10 in peripheral blood of lupus patients were mainly derived from monocytes. RNASE2 silencing in monocytes down-regulated IL-10 production and consequently reduced ABCs numbers in monocyte-B cell co-cultures, which could be restored by the addition of recombinant IL-10. Based on above findings, we concluded that RNASE2 might induce the production of ABCs *via* IL-10 secreted from monocytes, thus contributing to the pathogenesis of SLE.

## Introduction

Systemic lupus erythematosus (SLE) is a prototypic autoimmune disease characterized by the production of various autoantibodies and damage to multiple organ systems (1). It is widely recognized that B cell dysfunction plays a major role in the pathogenesis of lupus (2). Among which, the age-associated B cell subset (ABCs) has been the focus of increasing interest over the last decade (3). ABCs express myeloid markers CD11c and depend on T-box transcription factor (T-bet) for their generation, thus also called CD11c^+^T-bet^+^ B cells (4). ABC-like B cells have been detected in human SLE and animal models (5), and were associated with disease activity and specific autoantibody profiles (6). However, the molecular pathways that promote the expansion of ABC population in SLE patients is still largely unknown.

Recently, it has been recognized that the innate immune system not only initiates the inflammatory cascade in SLE, but also continues to promote adaptive immune response throughout the disease process (7, 8). Ribonuclease A family member 2 (RNase2), commonly known as eosinophil-derived neurotoxin, belongs to RNaseA superfamily and is one of the four major secretory proteins released upon activation of eosinophils (9). Besides eosinophils, human monocyte-derived macrophages can also produce this RNase after the stimulation (10). Our previous study has found that RNASE2 was one of the most prominent up-regulated genes in PBMCs of SLE patients through high-throughput sequencing technique (data not shown). Besides, RNASE2 has been identified as a marker gene that is cross-validated in multiple types of human SLE samples (11), while the mechanism by which this occurs remains obscure. Dependent on its ribonuclease activity, RNase2 has broad antiviral activity against single strand RNA like respiratory syncytial virus and human immunodeficiency virus (12, 13). Treatment of RNase2 could result in maturation of dendritic cells and trigger the production of a variety of soluble mediators, mostly pro-inflammatory cytokines and chemokines (14). More recently, it has been shown to be required in immune sensing of live pathogens by Toll-like receptor 8 (15). Thus, RNase2 may serve as a bridge between innate and adaptive immunity.

In this study, we demonstrated that RNASE2 mRNA was highly expressed in peripheral blood mononuclear cells (PBMCs) from SLE patients and correlated with disease activity, autoantibody levels as well as proportion of ABCs. Silencing RNASE2 reduced the number of ABCs *in vitro*. We also observed that interleukin (IL)-10 served as a major effector of RNASE2. By increasing the expression of IL-10 in monocytes, RNASE2 could promote the production of ABCs. Moreover, our data revealed that IL-10 has a two-way action on ABCs. Altogether, our study provides a novel mechanistic view into the upstream regulation of ABCs.

## Materials and Methods

### Subjects

Study protocol was reviewed and approved by the Ethics Committee of the Affiliated Drum Tower Hospital of Nanjing University Medical School. The enrollment of volunteers was conducted in compliance with the Declaration of Helsinki. Patients excluding tumors and infection are recruited from rheumatology and immunology department. Patients with SLE, rheumatoid arthritis (RA) and primary Sjögren’s syndrome (SS) fulfilled the 1997 updated American College of Rheumatology (ACR) classification criteria, the 1987 ACR criteria and the 2002 American-European consensus criteria respectively (16–18). SLE disease activity was assessed and scored by the SLE disease activity index (SLEDAI) and the British Isles Lupus Assessment Group (BILAG) index (19, 20). Written informed consent was obtained from all patients and healthy donors that provided blood samples 10ml or more specifically for the study. When only residual blood was used, written informed consent was waived.

### Cell Sorting

To simultaneously isolate multiple cell subsets from the same patient, flow cytometry sorting was applied. PBMCs were isolated from 10 ml peripheral venous blood with Ficoll-Hypaque discontinuous gradient method, donated by 5 HC and 8 SLE patients. PBMCs were stained with FITC-antiCD14 (BioLegend), eflour450-antiCD19 (ebioscience), PE-cyanine7-antiCD4 (ebioscience) antibodies, then flow cytometry sorting technology was applied: CD14^+^ gate and CD14^-^ gate were set on BD FACSAria III flow cytometer (BD Biosciences), CD14^+^ monocytes were separated; for CD14^-^ cells, CD19^+^ B cells and CD4^+^ T cells and the remaining CD14^-^CD19^-^CD4^-^ cells were isolated with CD4^+^ and CD19^+^ gates.

For B cell solo-cultures, B cell isolation kit (Miltenyi Biotec) was used to separate B cells from PBMCs. CD2, CD14, CD16, CD36, CD43, and CD235a (glycophorin A) positive cells were subsequently magnetically labeled with anti-Biotin microbeads for depletion. B cells obtained by depletion of magnetically labeled non-B cells were stained with percp cy5.5-antiCD19 (Biolegend), and flow cytometry showed the purity of CD19^+^ cells was over 90% (**Supplementary Figure 1A**).

For monocyte solo-cultures, human CD14 positive selection kit II (Stem cell) was applied to separate monocytes from lupus PBMCs according to the manufactory’s instructions. CD14^+^ monocytes were sorted using magnetic beads with a purity over 90% (**Supplementary Figure 2**).

### RNASE2 siRNA Silencing

Four pairs of small interfering RNA (siRNA) sequences targeting RNASE2 and one pair of non-targeting siRNA sequences (**Supplementary Table 1**) were designed and synthesized by personnel at Dharmacon Cells were suspended in siRNA buffer and distributed to 96-well round-bottomed plates (Costar) at 1×10^5^ per well, with RNASE2 siRNA pairs or nontargeting siRNA at a final concentration of 1uM. Cells were incubated at 37 °C with 5% CO_2_ for 3 days.

### Cell Cultures

PBMCs were isolated from peripheral venous blood with Ficoll-Hypaque discontinuous gradient method, donated by SLE patients. Then PBMCs were silenced by RNASE2 siRNA (1 uM, Dharmacon) for 3 days. Magnetic **s**orted B cells (1×10^5^) from SLE donors were stimulated with anti-CD40 (0.1 µg/ml, goat IgG, R&D Systems) and anti-IgM F(ab’)2 (5.0 µg/ml, Jackson ImmunoResearch Laboratories), and silenced by RNASE2 siRNA as described above. Magnetic sorted monocytes (Stem cell) were silenced as described above.

To confirm the role of monocytes on B cells and the link to RNASE2 and IL-10, 1×10^5^ monocytes sorted by Flow cytometry were treated with RNASE2 siRNA or anti IL-10 antibody (1 ug/ml and 5 ug/ml, Ebioscience) in 96 well plate with a volume of 100 ul for 6 hours, and then co-cultured with 0.5-1×10^5^/100 ul B cells from the same individual. For monocytes treated with RNASE2 siRNA, recombinant human IL-10 (50 ng/ml, Peprotech) was added in some of the co-cultures with B cells to observe the changes of ABCs.

To verify the effect of IL-10 on B cells, recombinant human IL-10 was either added into monocytes and B cells co-cultures at an increasing concentration of 0, 25, 50 or 100 ng/ml, or added into B cells solo-cultures stimulated with anti-CD40 and anti-IgM F(ab’)2 at low concentrations of 0, 2, 10 and 40 ng/ml without the presence of monocytes. Three days later, cells were harvested for examining by flow cytometry and culture supernatants were stored at -80 °C for use.

### RNA Isolation and Quantitative Real Time PCR

To detect RNASE2 and IL-10 expression, we isolated total RNA from PBMCs, CD19^+^ B cell, CD4^+^ T cell, monocytes and CD14^-^CD19^-^CD4^-^ cells using Trizol reagent (Vazyme). Contaminating DNA was removed by deoxyribonuclease treatment, and total RNA was quantified using spectrophotometry. The RNA was converted to complementary DNA (cDNA) using transcriptor first-strand cDNA kit (Vazyme), and qPCR was performed on StepOnePlus Real-Time PCR instrument with gene-specific primers and analyzed with StepOne SoftwareV2.3 (Applied Biosystems). The relative gene quantification was done by using the 2^−△△Ct^ method following normalization to glyceral-dehyde-3-phosphate dehydrogenase (GAPDH). For details of primers used in this study, see **Supplementary Table 2**.

### Flow Cytometry

For ABCs detecting, PBMCs/B cells to be detected were incubation with FcR blocking reagent (Miltenyi Biotec) for 10 minutes at 4 °C, then stained with BV421 anti-CD11c (BioLegend) and percp-Cy5.5 anti-CD19 (BioLegend) at 4 °C for 30 minutes. For intracellular staining, cells were surface stained, fixed using eBio fix/perm kit (eBioscience), washed and stained with PE-Cyanine7 anti-T-bet (BioLegend) in 1 × eBio fix/perm buffer for 1 hour at 4 °C. Mouse IgG1 kappa Isotype Control, PE-Cyanine7 (eBioscience) was used as isotype control for T-bet staining.

For RNASE2 detecting, PBMCs to be detected were incubation with FcR blocking reagent (Miltenyi Biotec) for 10 minutes at 4 °C, then stained with APC anti-CD14 and viability dye efour 506 (Invitrogen) at 4 °C for 30 minutes. For intracellular staining, cells were surface stained, fixed with fixation/permeabilization solution (Cytofix/Cytoperm kit, BD), washed and stained with RNASE2 antibody (Invitrogen) in 1× Perm/Wash buffer for 1 hour at 4 °C, then stained with AlexaFlour 488 Goat anti-rabbit IgG (FCMACS). Cells stained with CD14 and AlexaFlour 488 Goat anti-rabbit IgG were used as isotype control for RNASE2 staining.

Then Cells were analyzed on a BD FACSAria III flow cytometer (BD Biosciences) using FACSDiva software. Cells were analyzed on a BD FACSAria III flow cytometer (BD Biosciences) using Flowjo VX software.

### ELISA and Luminex

Total IgG and IL-10 levels in supernatants or plasma were detected by Human IgG total ELISA Kit (FMS-ELH102, Fcmacs) and Human IL-10 High Sensitivity ELISA Kit (70-EK110HS-96, MultiSciences). For total IgG level detection, samples and diluted standards were added to pre-embedded plate and incubated for two hours at 37 °C. Then biotinylated anti-human detection antibody was added to each well. The plate was incubated for one hour at 37 °C and developed with the addition of Streptavidin-HRP and tetramethylbenzidine as a substrate. The optical density for each well was documented with a microplate reader (Tecan Sunrise, Männedorf, Switzerland) set to 450 nm and levels of IgG and IL-10 were calculated according to their standard curves. For IL-10 level detection, amplification reagent was added to plate after the first addition of Streptavidin-HRP to amplify the detected signal. Levels lower than the lower limit of detection (LLOD) were regarded as having the minimum detection value (0.05 pg/ml).

B cell related cytokines was evaluated with customized MILLIPLEX MAP Human Th17 Magnetic Bead Panel (HTH17MAG-14K, Merck&Millipore) according to the manufacturers’ instructions, a kit used for the simultaneous quantification of the following cytokines: IL-2, IL-4, IL-6, IL-10, IL-12p70, IL-21, IFN-γ, TNF-α and TNF-β.

### Western Blot Analysis

Magnetic sorted 5×10^5^ CD14^+^ monocytes from SLE patients were silenced by RNASE2 siRNA (1uM, Dharmacon) for 3 days. Then cells were collected and proteins were extracted in RIPA buffer supplemented with EDTA-free protease inhibitor cocktail (Shanghai Epizyme) and phosphatase inhibitor cocktail (Shanghai Epizyme). Proteins were run on 12.5% gradient gel (BioRad), blotted onto PVDF membrane and detected with RNASE2 (Invitrogen) or GAPDH (Cell Signaling Technology) antibody. Images were captured and analyzed on Tanon−5200 Chemiluminescent Imaging System.

### Statistical Analysis

Statistical analyses were performed using GraphPad Prism 8.0.1 software. Data were shown as means and Standard Error of Mean (SEM). Unpaired two tailed t-test was applied to compare the difference between two groups, and Welch’s correction was applied for those with unequal variance. Paired two tailed t test was used for pairing data. Relations between two variables were evaluated using the Pearson correlation test or Spearman correlation test, dependent on whether the variables were normally distributed. A p value less than 0.05 was considered significant.

## Results

### Increased RNASE2 mRNA Expression in SLE Patients

**Figure 1:**
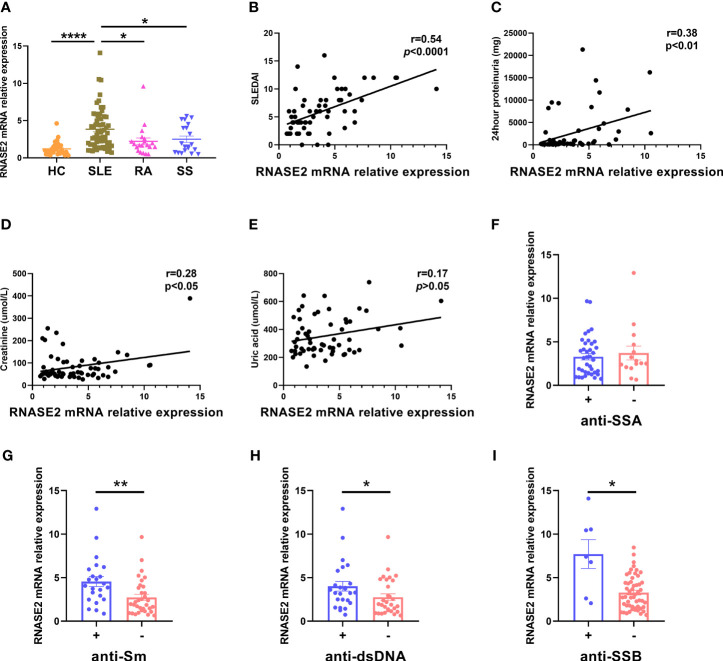
Increased RNASE2 mRNA expression in PBMCs from SLE patients. **(A)** Detection of RNASE2 expression by real-time PCR in PBMCs from 37 healthy controls (HC), 60 SLE patients, 20 patients with rheumatoid arthritis (RA) and 20 patients with primary Sjögren’s syndrome (SS). **(B–E)** Associations of RNASE2 mRNA levels with SLE disease activity index (SLEDAI) score, the amount of 24hour proteinuria as well as the levels of serum creatinine and uric acid by the Pearson or Spearman correlation test. **(F–I)** RNASE2 mRNA expression in SLE patients with or without positive anti-SSA, anti-Sm, anti-dsDNA or anti-SSB antibodies. Data are presented as mean ± SEM, *p < 0.05, **p < 0.01, ****p < 0.0001.

Elevated peripheral blood RNASE2 mRNA expression was validated by real-time PCR in 60 SLE patients, compared with 20 patients with rheumatoid arthritis (RA), 20 patients with primary Sjögren’s syndrome (SS) or 37 HC (**Figure 1A** and **Supplementary Table 3**). RNASE2 mRNA expression positively correlated with SLEDAI score, the amount of 24hour proteinuria, as well as creatinine (**Figures 1B–D**), but not uric acid (**Figure 1E**). Except for anti-SSA (**Figure 1F**), seropositive SLE patients for anti-Sm, anti-dsDNA or anti-SSB antibodies had increased RNASE2 levels (**Figures 1G–I**), implying a possible link between upregulated RNASE2 and autoantibody production.

### Association of ABC Proportion With RNASE2 Level

**Figure 2:**
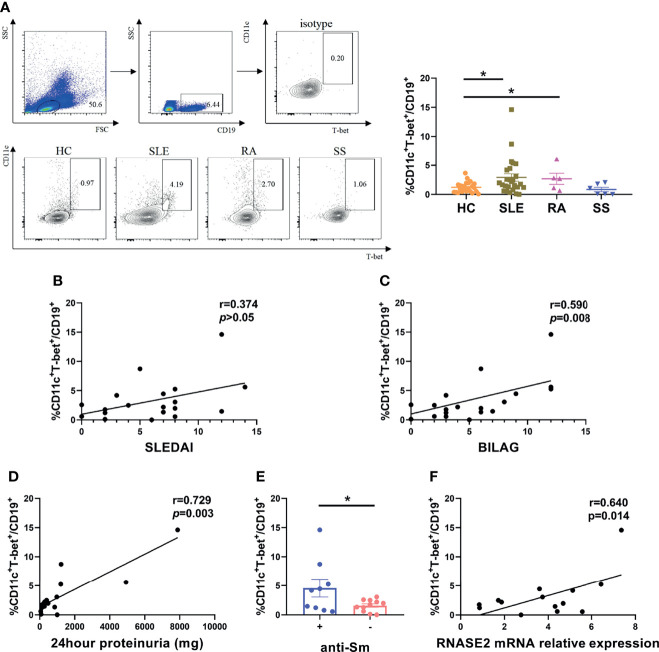
Association of age-associated B cells subset (ABCs) with RNASE2 expression in SLE patients. **(A)** PBMCs from 24 HC, 26 SLE, 5 RA and 6 SS patients was collected and analyzed for the proportion of CD11c^+^T-bet^+^ cells in CD19^+^ B cells by flow cytometry. **(B, C)** The percentage of CD11c^+^T-bet^+^ B cells was related to SLE disease activity score (SLEDAI and BILAG) (by Spearman correlation test). **(D)** The percentage of CD11c^+^T-bet^+^ B cells was associated with the amount of 24hour proteinuria (by Spearman correlation test). **(E)** The proportion of CD11c^+^T-bet^+^ B cells was increased in SLE patients with positive anti-Sm antibody. **(F)** The proportion of CD11c^+^T-bet^+^ B cells was closely related to RNASE2 mRNA levels in SLE patients (n=14). Data are presented as mean ± SEM, *p < 0.05.

The role of RNASE2 in SLE disease progression is unclear at present. Our data confirmed elevated proportion of peripheral ABCs in SLE patients (n=26) compared to that in HC (n=24) (**Figure 2A**), which was associated with both SLEDAI score and BILAG score as well as 24hour proteinuria levels (**Figures 2B–D**). Meanwhile, SLE patients with positive anti-Sm antibody also had higher levels of ABCs (**Figure 2E**). Given that RNASE2 and ABCs were both related to disease activities and autoantibodies production in SLE patients, it was speculated that these two factors might be connected. Then we detected peripheral RNASE2 expression by real-time PCR and ABCs proportion by flow cytometry simultaneously in another 14 SLE patients. Interestingly, a positive correlation between these two factors was truly observed (r=0.640, p=0.014) (**Figure 2F**).

### RNASE2 Silencing Down-Regulated The Proportion of ABCs

**Figure 3:**
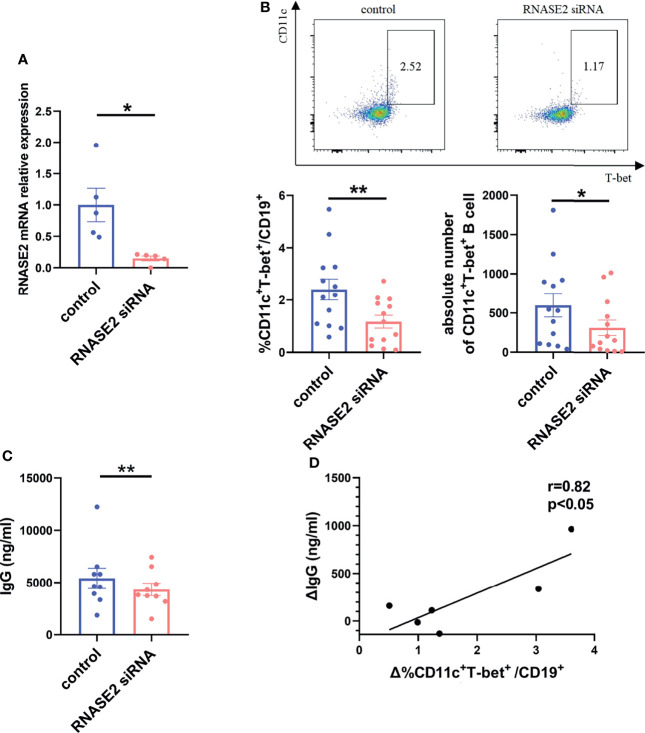
RNASE2 participated in the regulation of ABCs. **(A)** Nearly 80% reduction in the gene expression 3 days after the treatment of RNASE2 siRNA *in vitro* (n=5). **(B)** Decreased percentage and absolute number of CD11c^+^T-bet^+^ B cells after RNASE2 silencing by flow cytometry (n=13). **(C)** Down-regulated expression of total immunoglobulin (Ig) G in culture supernatants from RNASE2 silencing group (n=9), as determined by enzyme-linked immunosorbent assay (ELISA). **(D)** The difference of IgG level between silence group and control group was positively correlated to the alteration of ABCs proportion (n= 6). Data are presented as mean ± SEM, *p < 0.05, **p < 0.01.

To further prove the link between RNASE2 and ABCs, SLE PBMCs were cultured with RNASE2 small interfering RNA (siRNA) or non-targeting siRNA for 3 days. As showed in **Figure 3A**, there was an over 80% decrease of RNASE2 expression after silencing. Along with the decline of RNASE2, the proportion and absolute number of ABCs in PBMCs were both significantly reduced (**Figure 3B**), while the proportion and absolute number of total B cells in PBMCs showed no significant difference (**Supplementary Figure 3**). The levels of total immunoglobulin G (IgG) were also decreased after RNASE2 siRNA silence (**Figure 3C**), and the change of IgG levels was positively correlated with the range of ABCs reduction (r=0.82, p < 0.05) (**Figure 3D**), supporting that RNASE2 is involved in ABCs regulation and may consequently promotes antibody production.

### Reduced IL-10 Level After RNASE2 Silencing

**Figure 4:**
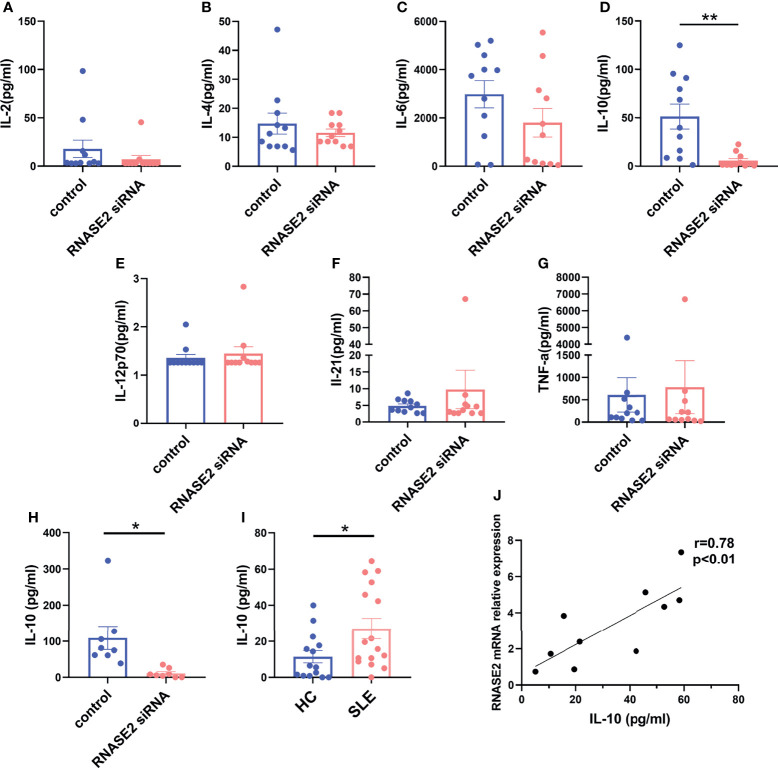
IL-10 was a major effector of RNASE2. **(A–G)** The expression of multiple cytokines (IL-2, IL-4, IL-6, IL-10, IL-12p70, IL-21 and TNF-α) in cultured supernatant was determined by using luminex liquid phase chip, among which only IL-10 level was significantly decreased in RNASE2 silencing group (n=11). The lower limit of detection (LLOD) for each cytokine was 2.96 pg/ml, 5.61 pg/ml, 0.72 pg/ml, 0.74 pg/ml, 1.26 pg/ml, 2.69 pg/ml and 0.98 pg/ml respectively, and levels lower than LLOD were regarded as having the minimum detection value. **(H)** Independent sample validation by ELISA confirmed the reduction of IL-10 level in cultured supernatants after RNASE2 silencing (n=8) (LLOD 0.05pg/ml). **(I)** *In vivo* study showed the level of plasma IL-10 was increased in SLE patients (n=16) compared with healthy controls (n=14). **(J)** Plasma IL-10 levels in SLE patients were closely linked to peripheral RNASE2 mRNA expression (n=10). Data are presented as mean ± SEM, *p < 0.05, **p < 0.01.

Next, we asked the question how ABCs were regulated by RNASE2. As RNASE2 was broadly connected to the production of cytokine mediators (14), the levels of B cell related cytokines in cultured supernatants after RNASE2 silencing were measured by using Luminex liquid phase chip technology, including IL-2, IL-4, IL-6, IL-10, IL-12p70, IL-21, interferon (IFN)-γ, tumor necrosis factor (TNF)-α and TNF-β. Our data showed undetectable levels of IFN-γ and TNF-β, and no difference in levels of IL-2, IL-4, IL-6, IL-12p70, IL-21 or TNF-α between RNASE2 silent group and control group (**Figures 4A–C, E–G**). As showed in **Figure 4D**, only IL-10 level was significantly decreased after RNASE2 silencing. In order to confirm this result, an independent sample validation test was applied by using ELISA and a nearly 90% decline of IL-10 was observed after RNASE2 silencing (**Figure 4H**). Consistently, we also found that plasma level of IL-10 in lupus patients were higher than those in normal subjects and positively correlated with peripheral RNASE2 expression (**Figures 4I, J**).

### IL-10 Mainly Derived From Lupus Monocytes

**Figure 5:**
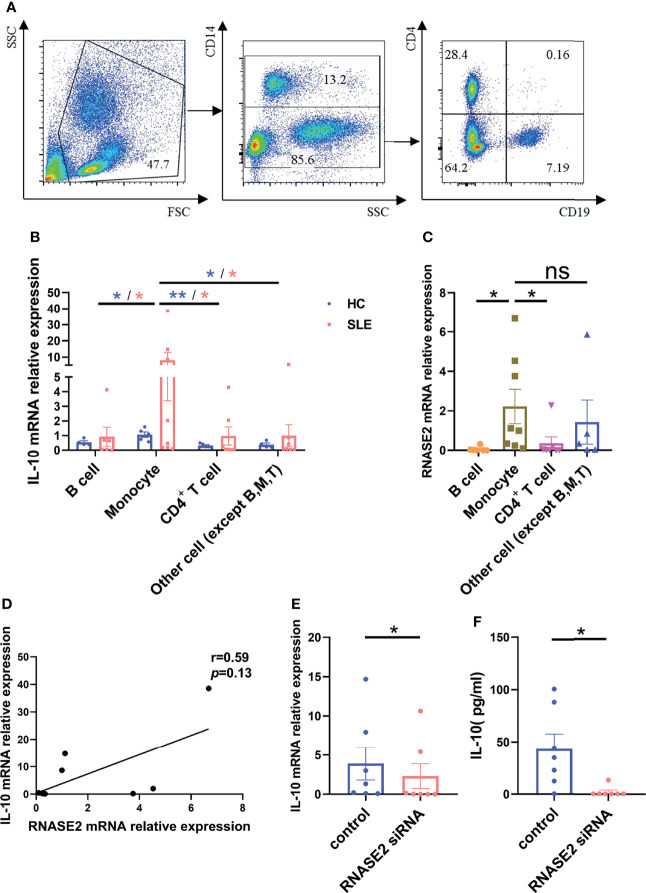
RNASE2 promoted the secretion of IL-10 from lupus monocytes. **(A)** Flow cytometry sorting technology was used to separate lupus CD14^+^ monocytes, CD19^+^ B cells, CD4^+^ T cells and the remaining CD14^-^CD19^-^CD4^-^ cells. **(B)** mRNA expression of IL-10 in each cell subgroup was detected by real-time PCR, and the highest expression was seen in monocyte subgroup (n=5), especially those from SLE patients (n=8). **(C, D)** mRNA expression of RNASE2 was also prominent in monocyte subgroup from lupus patients, which was correlated with monocyte IL-10 mRNA level (by Spearman correlation test, n=8). **(E, F)** RNASE2 silencing down-regulated IL-10 mRNA expression in lupus monocytes and IL-10 protein levels in cultured supernatants (n=7). Data are presented as mean ± SEM, *p < 0.05, **p < 0.01, ns, no significant statistical difference.

To search for the source of IL-10 in peripheral blood of SLE patients, we sorted out CD14^+^ monocytes, CD19^+^ B cells, CD4^+^ T cells and the remaining CD14^-^CD19^-^CD4^-^ cells from lupus PBMCs using flow cytometry sorting technology (**Figure 5A**), and measured the mRNA expression of IL-10 in each cell subgroup by real-time PCR. As showed in **Figure 5B**, similar to that in normal subjects, IL-10 expression in monocytes was highest among the groups in SLE patients. Meanwhile, RNASE2, usually highly expressed in eosinophils from normal subjects (**Supplementary Figure 4**), was also significantly elevated in lupus monocytes (**Figure 5C**). There was a trend towards significance between RNASE2 and IL-10 mRNA expression in SLE monocytes (r= 0.59) (**Figure 5D**). To verify the effect of RNASE2 on IL-10 secretion, we isolated CD14^+^ monocytes from lupus PBMCs by magnetic beads and cultured with RNASE2 siRNA. There was a significant decrease of RNSAE2 protein levels in SLE monocytes after siRNA treatment (**Supplementary Figure 5**). Levels of IL-10 mRNA and protein secretion in culture supernatants were both down-regulated after silencing of RNASE2 silencing (**Figures 5E, F**), suggesting that RNASE2 promotes IL-10 production in lupus monocytes.

### RNASE2 Regulated ABCs Through the Modulation of IL-10

**Figure 6:**
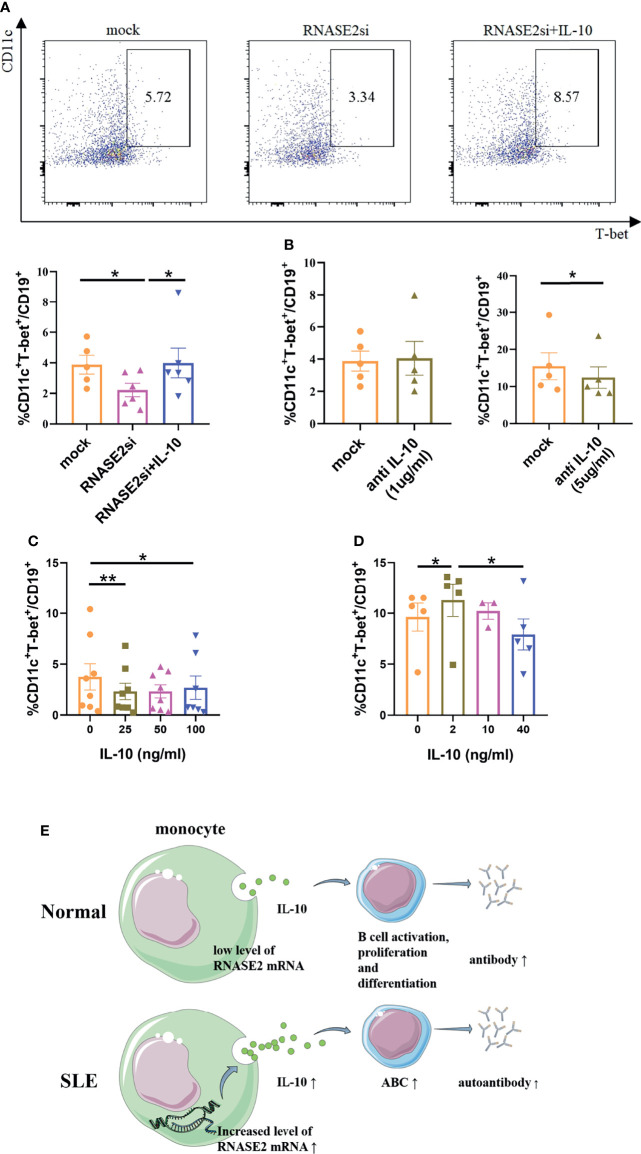
IL-10 regulated the production of ABCs in SLE patients. **(A)** Lupus B cells were co-cultured with monocytes with the presence of either RNASE2 siRNA (RNASE2si) or RNASE2 siRNA plus 50ng/ml human recombinant IL-10 (RNASE2si+IL-10) (n=6). The proportion of CD11c^+^T-bet^+^ B cells was restored after the replenishment of IL-10 in RNASE2 silencing group. **(B)** High dose anti-IL-10 (5ug/ml) but not low dose anti-IL-10 (1ug/ml) blocked the production of CD11c^+^T-bet^+^ B cells in B cell and monocyte co-cultures (n=5). **(C)** The effect of recombinant IL-10 on the proportion of CD11c^+^T-bet^+^ B cells in B-cell plus monocyte co-cultures (n=8). **(D)** The effect of recombinant IL-10 on the proportion of CD11c^+^T-bet^+^ B cells in CD19^+^ B cell culture (n=5). **(E)** Hypothesis schema of RNASE2 pathogenetic role involved in SLE patients (picture material was from http://smart.servier.com). Data are presented as mean ± SEM, paired t test, *p < 0.05, **p < 0.01.

To explore the relationship among RNASE2, IL-10 and ABCs, lupus B cells were negatively isolated from PBMCs by magnetic beads and cultured *in vitro* with or without the presence of RNASE2 siRNA, which showed no significant change in ABC percentages after RNASE2 silencing (**Supplementary Figure 1B**). Next, lupus monocytes and B cells were isolated respectively from SLE PBMCs. The monocytes were firstly silenced by RNASE2 siRNA for 6 hours, and then co-cultured with syngeneic B cells with or without human recombinant IL-10. As showed in **Figure 6A**, RNASE2 silencing significantly down-regulated the percentages of ABCs. While adding recombinant IL-10 to the co-culture fully restored ABCs levels after monocyte RNASE2 silencing, only high doses of anti-IL-10 antibody inhibited the production of ABCs in monocyte-B cell co-cultures (**Figure 6B**), suggesting that the presence of just a small amount of IL-10 in the culture system could promote the production of ABCs. Consequently, we identified a two-way action of IL-10: when IL-10 was added into monocyte-B cell co-cultures at relatively high concentration, the proportion of ABCs was actually down-regulated (**Figure 6C**), while only under low concentration (2ng/ml) and without the presence of monocytes, IL-10 promoted the expansion of ABCs (**Figure 6D**).

## Discussion

Recently, ABCs have attracted much attention in the pathogenesis of SLE. These CD11c^+^T-bet^+^ B cells are the main source of extra-follicular autoantibody production (6) and correlated with lupus manifestations, and their depletion *in vivo* may lead to reduction of autoreactive antibodies (21). In addition, excessive CD11c^+^T-bet^+^ B cells could promote activated T cells differentiated into follicular T-helper (Tfh) cells through their potent antigen-presenting function and consequently compromise affinity-based germinal center selection, participating in antibody-affinity maturation (22). However, despite cumulative data showing that ABCs are increased in SLE, current understanding regarding their expansion and regulation is rather limited. As reported, Tfh cells or T peripheral helper cells could provide help to ABCs *via* the secretion of IL-21 (23, 24). IL-21 acts synergistically with TLR7/9 to induce naive B cells differentiation into ABCs and promote T-bet expression, while IL-4 could inhibit the up-regulation of these cells and antagonize T-bet induction (25, 26). Besides, the SWEF proteins, a newly identified risk variant for human SLE, have also been found to be involved in ABCs regulation, which is dependent on cognate interactions with Tfh cells (27).

In this study, we showed for the first time that another upregulated gene in SLE, RNASE2, participated in the expansion of ABCs in the absence of T-cell help. Although RNASE2 has been recognized as a common marker gene associated with SLE (11), this gene does not receive much attention in recent years due to its unclear biological function. Normally, RNase2 is secreted by eosinophils and has broad antiviral activity (28). However, lupus monocytes appeared to be the most prominent cell type to produce this protein (**Figure 5C**). Lupus CD16^+^ monocytes are reported to have enhanced impacts on B cells to differentiate into plasma B cells with more Ig production, and less inhibition on regulatory B cells (29). The specific mechanism is unclear, which could only be partially attributed to the elevated surface expression of CD80, CD86 and HLA-DR on CD16^+^ monocytes. High expression of RNASE2 in lupus monocytes might contribute to promote B cell differentiation and Ig production.

Our next question is how RNase2 affected the function of lupus monocytes so as to promote ABCs expansion. Previously, we have tested the plasma levels of RNase2 and found there were no difference between SLE patients and healthy controls (data not shown), suggesting that RNase2 is less likely to act through secretion to distant target tissues in SLE pathogenesis, although it is one of the four major secretory proteins released upon activation of eosinophils under normal circumstances (30, 31). Since RNase2 could serve as a chemoattractant of dendritic cells and promote the secretion of a lot of cytokines and chemokines (14, 32), we focused on the measurement of several major cytokines related to B cells development. Surprisingly, we found that IL-10 but not IL-12 was the most effective cytokine to restore ABC levels after RNASE2 silencing, which was tightly associated with the decline of RNASE2 either in mRNA or in protein level.

Thus far, the role of IL-10 in SLE remains controversy. IL-10 can promote humoral immune responses, enhancing B cell proliferation, differentiation, and autoantibody production (33). Serum IL-10 levels have been reported to be increased in lupus patients and correlated with disease activity and anti-dsDNA antibodies (34). On the other hand, IL-10 is considered as a potent anti-inflammatory cytokine that can inhibit production of pro-inflammatory cytokines, antigen presentation, and cell proliferation (33, 35, 36). Our data support that IL-10 is generally pathogenic to promote the production of ABCs, yet it has the opposite effect at high concentrations. Also, it seems unrealistic to implement the corresponding antibodies to treat SLE patients, because there will be no efficacy until IL-10 is almost completely blocked.

It remains to be elucidated how RNASE2 regulates IL-10 production in lupus monocytes. Evidence has suggested that RNase2 is an endogenous ligand of TLR2. It appears to be a TLR2-specific as its capacity to enhance immune responses is independently of TLR1 or TLR6 (32). Recently, RNase2 has been found to act synergistically with RNase T2 to release uridine from RNA ligands, and help to process RNA into TLR8 ligands (15). Thus, through the stimulation of TLR, a lot of downstream signaling pathways, especially myeloid differentiation factor 88 (MyD88) (32) and mitogen-activated protein kinase (MAPK) (37), may be activated to promote the production of pro-inflammatory factors, including IL-10.

Based on the above data, a novel hypothesis of lupus pathogenesis has emerged: over-expression of RNASE2 in SLE patients may trigger monocytes to secrete more IL-10, consequently inducing the expansion of ABCs and leading to the production of various autoantibodies (**Figure 6E**). Therefore, RNASE2 may serve as a promising new target for the treatment of SLE.

## Data Availability Statement

The original contributions presented in the study are included in the article/**Supplementary Material**. Further inquiries can be directed to the corresponding author.

## Ethics Statement

The studies involving human participants were reviewed and approved by the Ethics Committee of the Affiliated Drum Tower Hospital of Nanjing University Medical School. The patients/participants provided their written informed consent to participate in this study, which was waived when only residual blood was used.

## Author Contributions

XF designed, coordinated and supervised the study. YZ drafted the manuscript. YZ, XT, and YX carried out most of the experiments, performed data acquisition and analysis. SW, WL, LG, and XM contributed to sample collecting and data interpretation. BT and LS participated in study design and helped revise the manuscript. All authors read and approved the final manuscript.

## Funding

This work is supported by National Natural Science Foundation of China (81971517, 81771745) and Jiangsu Provincial Medical Talent Program (ZDRCA2016059). LS was supported by Key Program of National Natural Science Foundation of China (81930043) and Major International (Regional) Joint Research Project of China (81720108020).

## Conflict of Interest

The authors declare that the research was conducted in the absence of any commercial or financial relationships that could be construed as a potential conflict of interest.

## Publisher’s Note

All claims expressed in this article are solely those of the authors and do not necessarily represent those of their affiliated organizations, or those of the publisher, the editors and the reviewers. Any product that may be evaluated in this article, or claim that may be made by its manufacturer, is not guaranteed or endorsed by the publisher.
