# Supplementary material for: RNASE2 Mediates Age-Associated B Cell Expansion Through Monocyte Derived IL-10 in Patients With Systemic Lupus Erythematosus
Source: Front Immunol. 2022 Feb 21;13:752189. doi: 10.3389/fimmu.2022.752189 (PMC8899663; doi:10.3389/fimmu.2022.752189)
Supplement: Supplementary file 1 [file DataSheet_1.pdf]

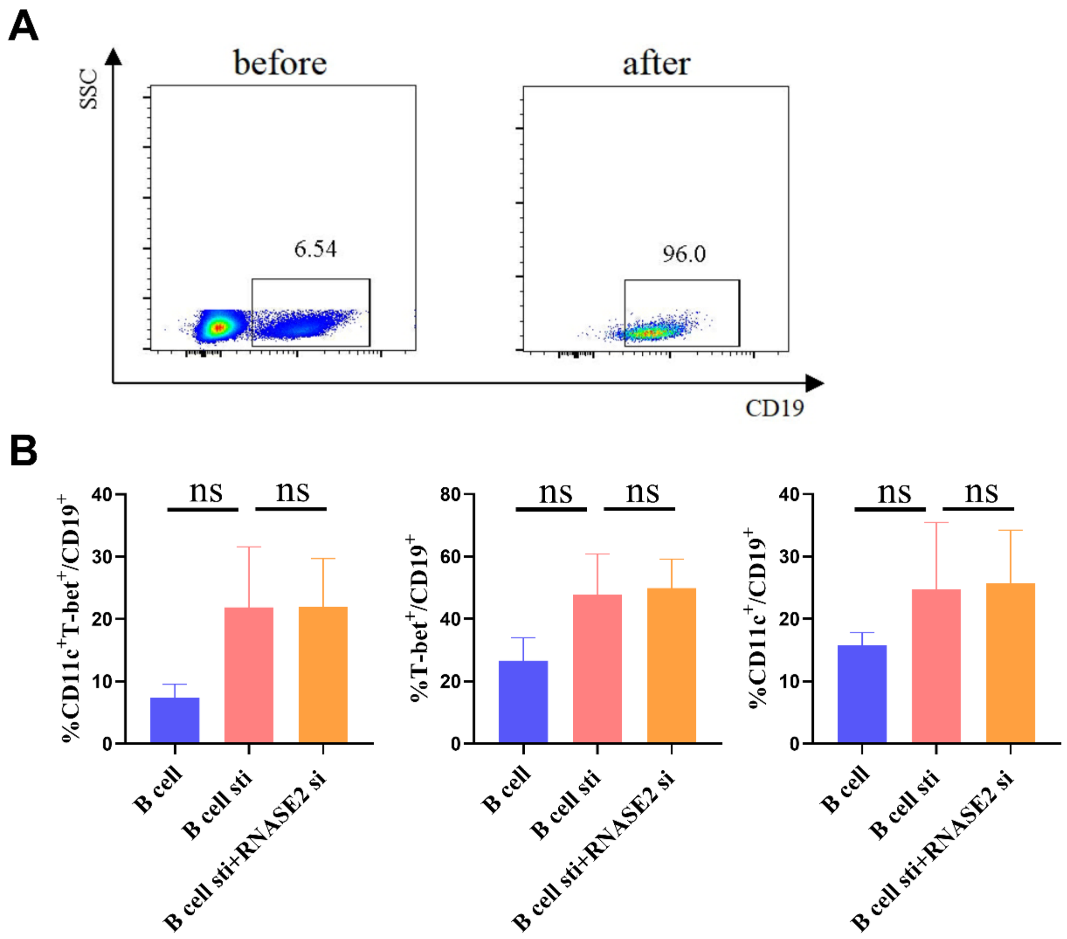


Supplementary figure 1. RNASE2 failed to modulate ABCs in B cell cultures. A, B cells were negative sorted from lupus PBMCs using magnetic beads, then $CD19^+$ B cells were verified up to 90% purity by flow cytometry. B, B cells were cultured with the stimulation of anti-CD40 and anti-IgM F(ab')2. Adding RNASE2 siRNA did not change the percentage of $CD11c^+$T-$bet^+$ B cells.

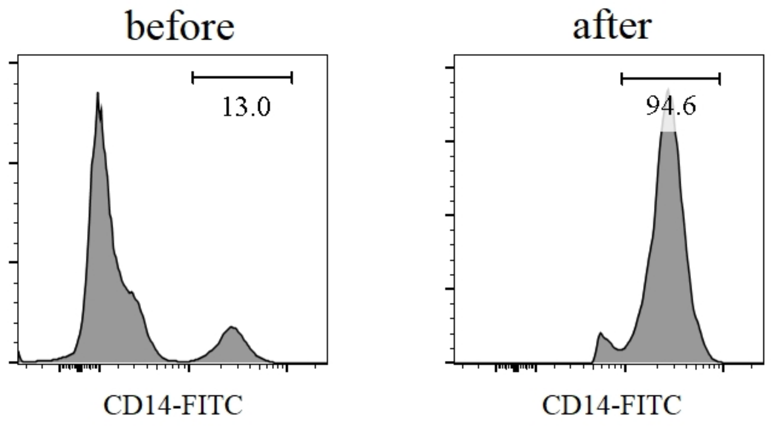


Supplementary figure 2. Purification of monocytes using magnetic beads. $CD14^+$ monocytes were positively sorted using magnetic beads, and an over 90% purity was detected by flow cytometry.

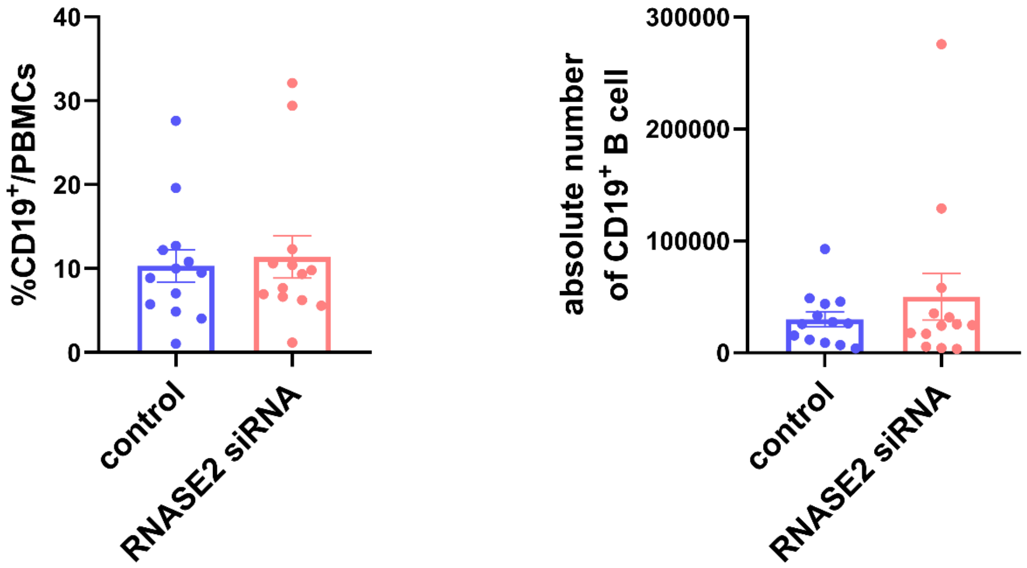


Supplementary figure 3. RNASE2 silence effect on total B cells. The proportion and absolute number of total B cells in PBMCs showed no significant difference after RNASE2 silencing by flow cytometry (n=13)

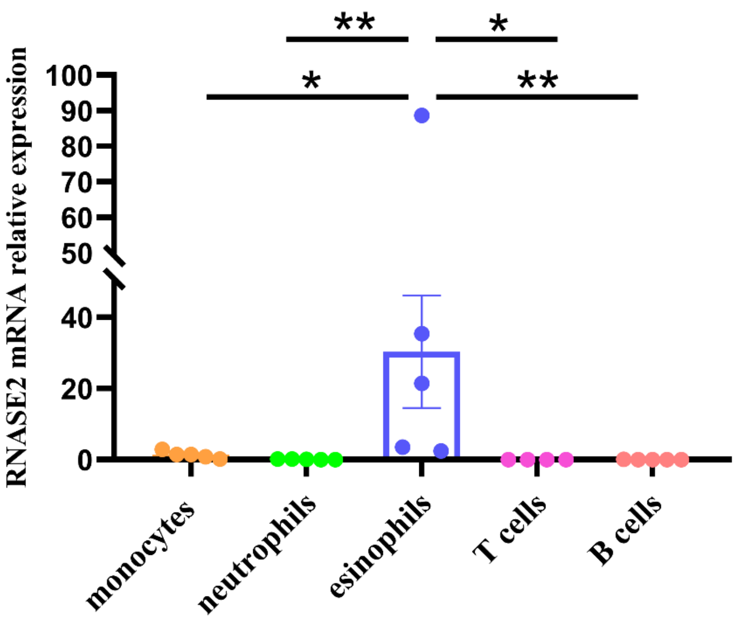


Supplementary figure 4. RNASE2 was mainly derived from eosinophils in normal subjects. The mRNA expression level of RNASE2 in monocytes ($CD45^{+}CD14^{+}$), neutrophils ($SSC^{high}CD45^{+}CD14^{-}CD16^{+}$), T cells ($CD45^{+}CD3^{+}$) and B cells ($CD45^{+}CD19^{+}$) were almost undetectable. *$p< 0.05$, **$p< 0.01$.

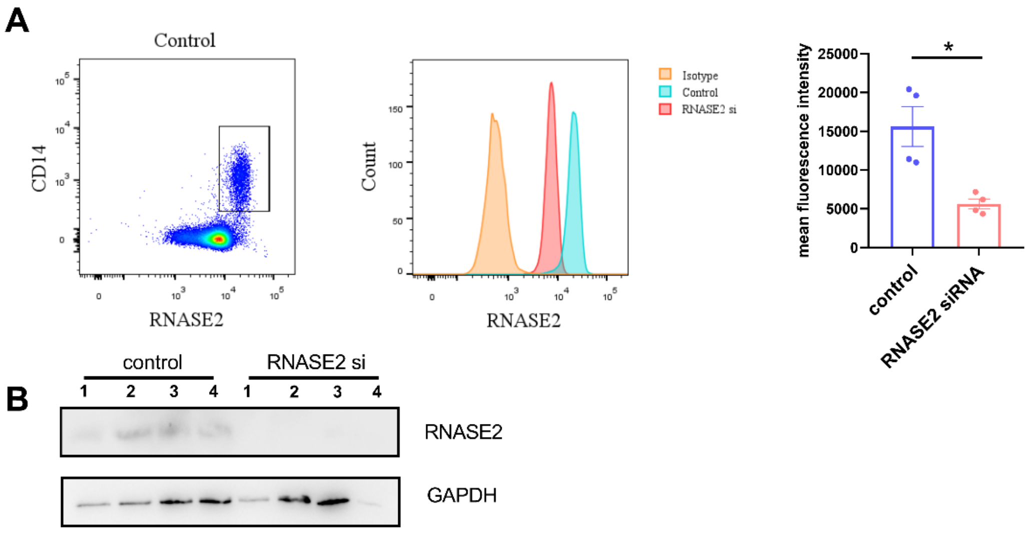


Supplementary figure 5. RNASE2 protein level decreased in SLE monocytes after RNASE2 silence. A, Decreased mean fluorescence intensity of RNASE2 protein level in $CD14^+$ monocytes after RNASE2 silence by flow cytometry (n=4). B， Reduced RNASE2 protein level after RNASE2 silence by western blot (n=4).

Supplementary table 1. siRNA sequences of RNASE2 and non-targeting control.

| | sense | antisense |
|---|---|---|
| RNASE2-1 | 5'-GACCUGUCCUAGUAACAAA-3' | 5'-UUUGUUACUAGGACAGGUC-3' |
| RNASE2-2 | 5'-CCCAUCUCUCCAUAUACUU-3' | 5'-AAGUAUAUGGAGAGAUGGG-3' |
| RNASE2-3 | 5'-UACCUGGGCUCAAUGGUUU-3' | 5'-AAACCAUUGAGCCCAGGUA-3' |
| RNASE2-4 | 5'-CAGUUCACCUGGAUAGAAU-3' | 5'-AUUCUAUCCAGGUGAACUG-3' |
| Non-targeting siRNA | 5'-UUCUCCGAACGUGUCACGU-3' | 5'-ACGUGACACGUUCGGAGAA-3' |

Supplementary table 2. Primers for mRNA detection.

| gene | GenBank number | Primer sequence |
|---|---|---|
| RNASE2 | NM_002934 | F：5'-ACCTCACAACTCCAAGTCCA-3' |
| | | R：5'-GGATACTGTGGAGGGTCTCG-3' |
| IL-10 | NM_000572.3 | F：5'-TACGGCGCTGTCATCGATTT-3' |
| | | R：5'-ACTCATGGCTTTGTAGATGCCT-3' |
| GAPDH | NM_002046 | F：5'-ATGGGGAAGGTGAAGGTCG-3' |
| | | R：5'-GGGGTCATTGATGGCAACAATA-3' |

Supplementary table 3. Demographics of the patients.

| Characteristics | SLE (n=60) | RA (n=20) | SS (n=20) |
|---|---|---|---|
| Age, mean±SD (years) | 39.5±13.3 | 58.4±14.0 | 46.9±13.0 |
| Sex, no. male/female | 4/56 | 3/17 | 1/20 |
| Disease duration （months） | 8.7±9.0 | — | — |
| SLEDAI score | 6.1±4.0 | — | — |
| BILAG score | 5.3±3.7 | — | — |
| Clinical Characteristics | | | |
| Malar Rash | 35(58.3%) | — | — |
| Photosensitivity | 15 (25%) | — | — |
| Oral ulcers | 8 (13.3%) | — | — |
| Arthritis | 34 (56.7%) | — | — |
| Pleuritis or Pericarditis | 10 (16.7%) | — | — |
| Renal Disorder | 45 (75%) | — | — |
| Neurologic Disorder | 2 (3.3%) | — | — |
| Hematologic Disorder | 13 (21.6%) | — | — |
| Laboratory test | | | |
| WBC, mean±SD (x10^9/L) | 6.5±1.7 | — | — |
| IgG, mean±SD (g/L) | 10.0±2.6 | — | — |
| IgM, mean±SD (g/L) | 1.0±0.2 | — | — |
| C3, mean±SD (g/L) | 0.9±0.3 | — | — |
| C4, mean±SD (g/L) | 0.1±0.0 | — | — |
| ESR (mm/h) | 41.8±16.8 | — | — |
| CRP (mg/L) | 31.7±15.1 | — | — |
| Urine protein (mg/24h) | 1067.0±398.4 | — | — |
| Positive ANA, n (%) | 56(93.3%) | — | — |
